# Leucine supplementation combined with resistance exercise improves the plasma lipid profile of dexamethasone-treated rats

**DOI:** 10.1186/1476-511X-11-7

**Published:** 2012-01-11

**Authors:** Humberto Nicastro, Claudia R da Luz, Daniela FS Chaves, William das Neves, Kerolyn S Valente, Antonio H Lancha

**Affiliations:** 1 Laboratory of Applied Nutrition and Metabolism, Physical Education and Sports School, University of São Paulo - São Paulo, SP, Brazil

**Keywords:** Branched-chain amino acids, glucocorticoid, lipid profile, exercise

## Abstract

The impact of leucine supplementation and resistance exercise (RE) on plasma lipid profile was evaluated in adult rats treated with dexamethasone, an experimental model of dyslipidemia. Total cholesterol did not differ among groups. Furthermore, leucine supplementation did not promote improvement in the plasma total cholesterol and LDL-c of the animals. However, plasma TG and VLDL-c were significantly decreased and HDL-c increased after 7 days of leucine supplementation combined with RE. In conclusion, leucine supplementation combined with RE, but not isolated, improved the plasma lipid profile of dexamethasone-induced dyslipidemic rats.

## Introduction

Glucocorticoids are widely prescribed drugs in clinical practice (chronic rheumatic diseases) due to their potent anti-inflammatory effects. However, low dose/long term or high dose/short term use are known to induce severe adverse metabolic effects, such as dyslipidemia due to elevated serum cortisol levels [1].

In contrast, leucine supplementation has demonstrated potential therapeutic effects preventing obesity and significantly modulating lipid profile in animals exposed to high-fat feeding [2,3]. Corroborating such therapeutic effects, mechanical stimuli promoted by resistance exercise (RE) has also been considered a potent non-pharmacological tool. Muscle contraction may enhance blood glucose uptake by skeletal muscle through insulin-independent mechanisms [4] and improve serum lipid profile of dyslipidemic women [5].

We aimed to evaluate the effects of two therapeutic strategies on dexamethasone-induced dyslipidemia in rats: leucine supplementation and RE. To our knowledge, this is the first report evaluating the effects of such combined interventions on dexamethasone-treated rats.

## Materials and methods

Animal's care, dexamethasone treatment, nutritional supplementation, and the RE protocol have been previously described [6,7]. All methods used were approved by the Local Ethical Committee for Animal Research.

Briefly, Wistar male rats (400-450 g) were housed under controlled environmental conditions with free access to food and water before the experiments were performed. Rats were randomly divided into the following groups: 1) dexamethasone (DEX; n = 06), 2) control pair-fed (CON-PF; n = 06), 3), 4) dexamethasone plus leucine (DEX-LEU; n = 07), dexamethasone plus RE (DEX-RE; n = 05) and dexamethasone plus leucine and RE (DEX-LEU-RE; n = 05). During 7 days, dexamethasone (5 mg·kg·day^-2^) was given daily via drinking water. Leucine-supplemented groups received 0.135 g·kg·day^-2 ^through gavage (at 09:00 a.m.). Trained rats performed 3 sessions of a squat-type exercise in one daily session with 2 days of rest interval between sessions [7]. All groups were pair-fed to the DEX-treated group according to individual body weight. Animals were killed by decapitation after an overnight fast of 12 hours.

Plasma lipoproteins concentration (total cholesterol, triglycerides - TG, low-density lipoprotein - LDL-c, high-density lipoprotein - HDL-c) were measured using enzymatic kits (BioTécnica^®^, São Paulo, Brazil). Very low-density lipoprotein (VLDL-c) was calculated using Friedewald's equation as follows: VLDL-c = TG/5.

The results are expressed as mean ± SEM. The dependent variables were tested by analysis of variance (ANOVA) one-way (treatment) and a post hoc test with a Tukey adjustment was performed for multiple comparison purposes. The significance level was set at p < 0.05.

## Results and Discussion

**Figure 1:**
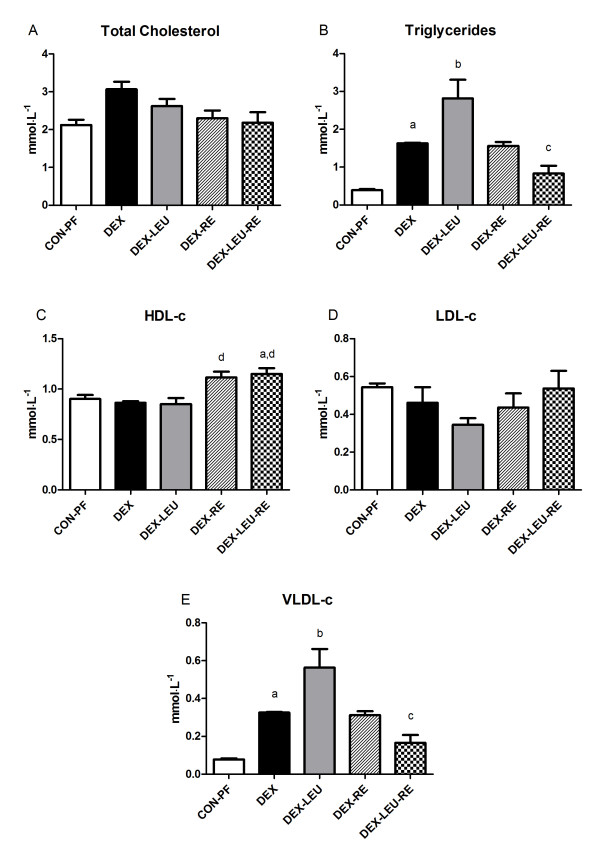
**Plasma lipid profile of dexamethasone-treated rats submitted to leucine supplementation and RE**. HDL-c, high-density lipoprotein; LDL-c low-density lipoprotein; TG, triglycerides; VLDL-c, very low-density lipoprotein. Data are expressed as mean ± SEM. ^a ^p < 0.05 vs. CON-PF; ^b ^p < 0.001 vs. CON-PF; ^c ^p < 0.01 vs. DEX-LEU; ^d ^p < 0.01 vs. CON-PF; ^e ^p < 0.05 vs. DEX-LEU.

Figure 1 shows the plasma lipid profile of the experimental groups. We observed that plasma total cholesterol and LDL-c did not differ among groups (p > 0.05), suggesting that, in our experimental model, dexamethasone-induced imbalance in plasma lipid profile did not reflect in total cholesterol and LDL-c and that leucine supplementation and RE were not able to modulate it. Although not significant, DEX group presented ~24% of increase in plasma total cholesterol compared to the CON-PF group.

In contrast, plasma TG and VLDL-c were significantly increased in DEX group when compared to the CON-PF group (p < 0.05). Leucine supplementation did not worse these plasma lipoproteins when compared to the DEX group (p > 0.05), but increased them to a greater extent than DEX group when compared to the CON-PF group (p < 0.001). These data demonstrate that leucine supplementation may act as a dexamethasone synergist in induction of dyslipidemia. However, DEX-LEU-RE group showed significantly reduced plasma TG and VLDL-c when compared to the DEX-LEU group (p < 0.01). Since the DEX-RE group presented no significant effect in plasma TG and VLDL-c when compared to the DEX group (p > 0.05), it is possible to suggest that leucine supplementation and RE present therapeutic effects on dexamethasone-induced dyslipidemia only when they are applied together. According to the data presented, it is possible to speculate that leucine supplementation enhances the effect of muscle contraction.

Corroborating these therapeutic effects, plasma HDL-c was also significantly increased in DEX-LEU-RE group when compared to the DEX-LEU group (p < 0.05). It is interesting to note that DEX-RE and DEX-LEU-RE groups showed increased plasma HDL-c in comparison to the CON-PF-group (p < 0.01 and p < 0.05, respectively). Such data provide us two main conclusions: 1) leucine supplementation combined with RE is definitely a therapeutic nutritional strategy in attenuating dexamethasone-induced dyslipidemia and 2) even in the presence of dexamethasone, RE improved plasma HDL-c when compared to basal condition.

In conclusion, leucine supplementation combined with RE, but not isolated, promoted significant improvements in the plasma lipid profile of dexamethasone-induced dyslipidemia in rats. Such strategy can represent an interesting nutritional strategy able to promote cardiovascular benefits [8].

## Abbreviations

HDL-c: high-density lipoprotein; HOMA-IR: homeostasis model for assessment of insulin resistance; LDL-c: low-density lipoprotein; RE: resistance exercise; TG: triglycerides; VLDL-c: very low-density lipoprotein.

## Competing interests

The authors declare that they have no competing interests.

## Authors' contributions

HN conceived and designed the study, participated in data collection and analyses and drafted the manuscript; CRL and DFSC participated in the data collection and analyses and helped to draft the manuscript; WN and KSV participated in the data analyses and helped to draft the manuscript; AHL Jr participated in the design of the study and drafted the manuscript. All authors read and approved the final manuscript.

## Authors' information

HN is a Ph.D. student; CRL is a M.Sc. student; DFSC is a post-doc student; WN and KVSC are scientific initiation students; AHL Jr is full professor of Nutrition and Metabolism.
